# Breakfast Skipping in Female College Students Is a Potential and Preventable Predictor of Gynecologic Disorders at Health Service Centers

**DOI:** 10.3390/diagnostics10070476

**Published:** 2020-07-13

**Authors:** Tomoko Fujiwara, Masanori Ono, Takashi Iizuka, Naomi Sekizuka-Kagami, Yoshiko Maida, Yumi Adachi, Hiroshi Fujiwara, Hiroaki Yoshikawa

**Affiliations:** 1 Department of Social Work and Life Design, Kyoto Notre Dame University, 1 Minami-Nonogami-cho, Shimogamo, Sakyo-ku, Kyoto 606-0847, Japan; 2 Department of Obstetrics and Gynecology, Graduate School of Medical Science, Kanazawa University, Kanazawa 920-8640, Japan; masanori@med.kanazawa-u.ac.jp (M.O.); zukatti@staff.kanazawa-u.ac.jp (T.I.); fuji@med.kanazawa-u.ac.jp (H.F.); 3 Department of Nursing, College of Medical, Pharmaceutical, and Health Sciences, Kanazawa University, Kanazawa 920-8640, Japan; sekky@mhs.mp.kanazawa-u.ac.jp (N.S.-K.); maida@staff.kanazawa-u.ac.jp (Y.M.); 4 Health Service Center, Kanazawa University, Kanazawa 920-1192, Japan; adachiy@staff.kanazawa-u.ac.jp (Y.A.); hiroaki@staff.kanazawa-u.ac.jp (H.Y.)

**Keywords:** ADHOGD, adolescent, breakfast skipping, diet, dietary habits, dysmenorrhea, endometriosis, oral contraceptives, young adulthood

## Abstract

Inadequate dietary habits in youth are known to increase the risk of onset of various diseases in adulthood. Previously, we found that female college students who skipped breakfast had higher incidences of dysmenorrhea, suggesting that breakfast skipping interferes with ovarian and uterine functions. Since dietary habits can be managed by education, it is preferable to establish a convenient screening system for meal skipping that is associated with dysmenorrhea as part of routine services of health service centers. In this study, we recruited 3172 female students aged from 18 to 25 at Kanazawa University and carried out an annual survey of the status of students’ health and lifestyle in 2019, by a questionnaire. We obtained complete responses from 3110 students and analyzed the relationship between dietary habits, such as meal skipping and history of dieting, and menstrual disorders, such as troubles or worries with menstruation, menstrual irregularity, menstrual pain, and use of oral contraceptives. The incidence of troubles or worries with menstruation was significantly higher in those with breakfast skipping (*p* < 0.05) and a history of dieting (*p* < 0.001). This survey successfully confirmed the positive relationship between breakfast skipping and menstrual pain (*p* < 0.001), indicating that this simple screening test is suitable for picking up breakfast skippers who are more prone to gynecologic disorders. In conclusions, since dysmenorrhea is one of the important clinical signs, breakfast skipping may become an effective marker to predict the subsequent onset of gynecological diseases at health service centers. Considering educational correction of meal skipping, breakfast skipping is a potential and preventable predictor that will contribute to managing menstrual disorders from a preventive standpoint in the future.

## 1. Introduction

Poor dietary behaviors in youth were reported to increase the risk of onset of lifestyle diseases in adulthood. [1]. Consequently, both insufficient energy intake and inadequate timing of food intake are considered to be current nutritional issues during young adulthood [2,3]. At the beginning of college life, since considerable numbers of students start living alone, their dietary habits can become poor during the post-adolescent stage, in Japan [4]. For example, rates of meal-skipping and dieting were reported to be high during young adulthood [3,5]. On the other hand, it is widely accepted that excess dieting induces gynecological disorders, such as dysmenorrhea, irregular menstruation, and hypothalamic amenorrhea [6,7,8,9]. In addition, recent studies proposed that endometriosis, which is frequently manifested by dysmenorrhea, latently develops as a consequence of modern dietary lifestyles [10], suggesting that gynecological disorders are closely related to dietary habits [11].

Based on this background, we found that female college students who skipped breakfast had a significantly higher incidence of dysmenorrhea on a questionnaire-based investigation [12]. We also observed that the incidence of irregular menstruation was high in a breakfast-skipping population [13], indicating that skipping breakfast is associated with ovarian and uterine dysfunctions in young women [7]. From these findings, we hypothesize that breakfast skipping disrupts the hypothalamic–pituitary function and impairs the reproductive rhythm by disrupting central clock system, leading to ovarian and uterine dysfunction [13]. Since the incidence of dysmenorrhea is high in female college students with a history of dieting, we considered that inadequate dietary habits in adolescence induce the subsequent development of organic gynecologic diseases [14], and named this concept “adolescent dietary habit-induced obstetric and gynecologic disease (ADHOGD)” [15].

Since dietary habits in young adulthood can be managed by education, the concept of ADHOGD may contribute to developing prophylaxes for female reproductive diseases, changing the focus from a therapeutic approach in a hospital to a prophylactic one in health service centers of universities [15]. The initial step to achieve prophylaxis is to establish a convenient screening system for meal skipping that is associated with dysmenorrhea. It is preferable for such a screening test to be feasible as a part of routine services of health service centers. Therefore, in this study, we modified the annual health questionnaire investigation of Kanazawa University by adding several questions, and statistically analyzed the relationship between dietary habits and menstrual disorders.

## 2. Materials and Methods

### 2.1. Subjects

**Figure 1:**
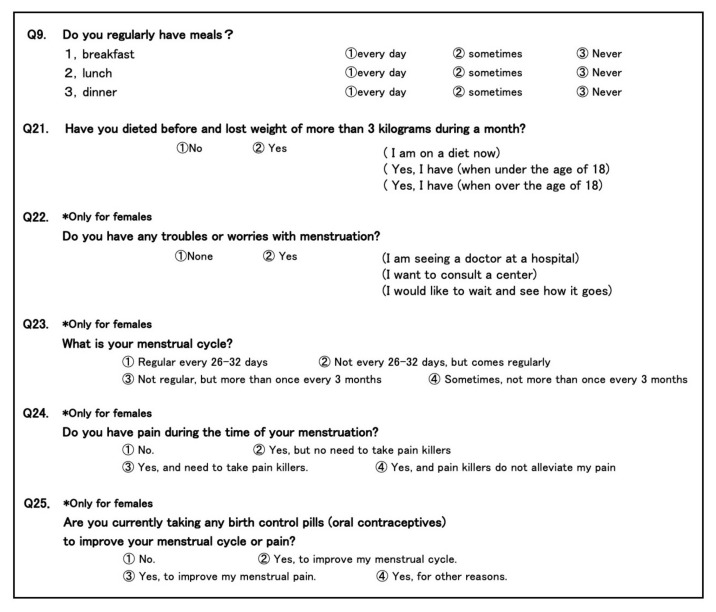
The contents of a survey sheet. Food-frequency information about breakfast, lunch, and dinner is obtained by question no. 9. History of dieting is strictly defined as those who experienced more than 3 kg reduction of body weight during a month, and this item was added to the survey sheet (question no. 21). For menstrual disorders, four questionnaire entries: troubles or worries with menstruation, menstrual irregularity, menstrual pain, and use of oral contraceptives, are prepared in question nos. 22–25.

The subjects were young Japanese women aged from 18 to 25 years old who studied in Kanazawa University. The study protocol was approved by the Medical Ethics Committee at Kanazawa University (no. 2011-113 (17-12), the 12th revised protocol was approved on 2018/11/01). We carried out an annual survey of the status of students’ health and lifestyle at the beginning of the school year in 2019 by a questionnaire, which was performed in order to support and improve the health status and lifestyles of students on campus. Information regarding the aim of this survey was presented with the questionnaire. The total number of participants in 2019 was 3172, and we obtained complete responses that were suitable for statistical analysis from 3110 female students. The contents of the survey sheet are shown in Figure 1.

### 2.2. Dietary Habits

#### 2.2.1. Meal Skipping

Simple food-frequency questionnaires about breakfast, lunch, and dinner of the previous surveying sheet were used without any change and all participants were divided into three groups: Group I: breakfasting every day; Group II: breakfasting sometimes; and Group III, having no breakfast (question no. 9 in Figure 1).

#### 2.2.2. History of Dieting

In this study, history of dieting was strictly defined as those who experienced more than 3 kg reduction of body weight during a month, and this item was added to the survey sheet (question no. 21 in Figure 1). All participants were divided into two groups: Group I: history absent, and Group II: history present. Group II was further sub-divided into three groups: Group A: dieting at present, Group B: dieted before 18 years old and Group C: dieted after 18 years old.

### 2.3. Menstrual Disorders

For menstrual disorders, we added four questionnaire entries: troubles or worries with menstruation, menstrual irregularity, menstrual pain, and use of oral contraceptives, to the survey sheet, as shown in question nos. 22–25 in Figure 1.

#### 2.3.1. Troubles or Worries with Menstruation

As for troubles or worries with menstruation, we simply confirmed their presence, and all participants were divided into two groups: Group I: absent, and Group II: present (question no. 22 in Figure 1). Group II was further sub-divided into three groups: Group A: seeing a doctor at a hospital, Group B: want to consult a center, and Group C: want to wait and see how it goes.

#### 2.3.2. Menstrual Irregularity

Regular menstruation was defined as constant 26–32-day intervals in each menstrual cycle. Menstrual irregularity was classified into four groups (question no. 23 in Figure 1): Group I: regular, Group II: periodically constant, but not within 26–32-day intervals, Group III: irregular, but within 3 months, and Group IV: irregular, sometimes more than 3 month-intervals. These 4 groups were subjected to scoring at 4 stages and the differences were analyzed by the Kruskal–Wallis test.

#### 2.3.3. Menstrual Pain

Menstrual pain was classified into four categories (question no. 24 in Figure 1) according to the previous reports [12,13]: Group I: free of pain, Group II: painful, but can manage without analgesic, Group III: painful, requiring analgesic, and Group IV: painful, not relieved by analgesic. These 4 groups were subjected to scoring at four stages and the differences were analyzed by the Kruskal–Wallis test.

#### 2.3.4. Use of Oral Contraceptives

Although the numbers of oral contraceptive users are relatively limited in Japan, we observed their use and reasons, to clarify the background of oral contraceptive users. Since oral contraceptives are effective for the management of menstrual irregularity and pain [16], such information is useful to correct the data concerning students whose menstrual disorders have been improved by oral contraceptives. All participants were divided into four groups: Group I: not using, Group II: using to improve menstrual cycle, Group III: using to improve menstrual pain, and Group IV: using for other reasons (question no. 25 in Figure 1).

### 2.4. Statistical Analysis

Differences in irregular menstruation and menstrual pain among the groups with different dietary habits were calculated by the Kruskal–Wallis test, followed by the Mann–Whitney test for multiple comparisons. The relationship of dietary habits with the incidence of troubles or worries with menstruation or use of oral contraceptives was analyzed by the chi-square test and Ryan procedure as a post-hoc test [17]. *P*-values less than 0.05 were considered significant. Logistic regression analysis was further performed to adjust potential confounders and calculate the odds ratio of the factors showing significant differences.

## 3. Results

### 3.1. Dietary Habits

#### 3.1.1. Meal Skipping

The numbers of those breakfast skipping in Groups I (every day), II (sometimes), and III (none) were 2101 (67.6%), 881 (28.3%), and 128 (4.1%), respectively. The numbers of those lunch skipping in the three groups were 2936 (94.41%), 173 (5.56%), and 1 (0.03%), whereas those dinner skipping numbered 2917 (93.79%), 191 (6.14%), and 2 (0.06%), respectively.

#### 3.1.2. History of Diet

The numbers of those with a history of dieting were 2919 (93.9%) in Group I (none) and 191 (6.1%) in Group II (done). Twenty-four students of Group II failed to answer the subsequent sub-questionnaires. The numbers of those in Groups A (at present), B (before 18 years old), and C (after 18 years old) were 12 (7.2%), 82 (49.1%), and 73 (43.7%), respectively.

### 3.2. Menstrual Disorders

#### 3.2.1. Troubles or Worries with Menstruation

The number of students having troubles or worries with menstruation was 549 (17.7%), whereas that of students without them was 2561 (82.3%, Table 1). When we classified meal skipping groups into two groups, having every day and not having every day, the incidence of troubles or worries with menstruation was significantly higher in those with habits of breakfast and lunch skipping by the chi-square test (*p* < 0.01 and *p* < 0.05, respectively), whereas the difference was not significant in dinner skipping. The incidence of menstrual troubles or worries was significantly higher in the group with a history of dieting by the chi-square test (*p* < 0.001). When we adjusted potential confounders by logistic regression analysis, the significance of lunch skipping disappeared (Table 2).

Eleven students of Group II failed to answer the subsequent sub-questionnaires. The numbers of those in Groups A (seeing a doctor), B (want to have consultation), and C (want to wait) were 205 (38.1%), 18 (3.3%), and 315 (58.6%), respectively.

#### 3.2.2. Menstrual Irregularity

The numbers of students in Groups I (regular), II (constant, out of 26–32-day interval), III (irregular, within 3 months), and IV (sometimes, more than 3-month interval) were 2091, 578, 379, and 62, respectively (Table 3). Significant differences between menstrual irregularity and meal skipping or history of dieting were not detected.

#### 3.2.3. Menstrual Pain

The numbers of students in Groups I (free of pain), II (not requiring analgesic), III (requiring analgesic), and IV (not relieved by analgesic) were 683, 1214, 1157, and 56, respectively (Table 4). A significant correlation between breakfast skipping and menstrual pain was observed by the Kruskal–Wallis test, followed by the Mann–Whitney test for multiple comparisons (*p* < 0.001). In contrast, no significant correlation of menstrual pain with lunch or dinner skipping was observed. There was also no significant correlation between menstrual pain and history of dieting. After meal having patterns were classified into two groups, having every day and not every day, we performed a logistic regression analysis and obtained the similar results (Table 2).

#### 3.2.4. Use of Oral Contraceptives

The number of oral contraceptive users was 140 (4.5%). Among them, the numbers used for menstrual cycle control, menstrual pain, and other reasons were 59 (42.1%), 56 (40.0%), and 25 (17.9%), respectively (Table 5). A significant correlation between history of dieting and use of oral contraceptives was observed (*p* < 0.001). In contrast, there was no significant correlation between use of oral contraceptives and meal skipping.

#### 3.2.5. Correction by Data of Oral Contraceptive Users

Although the rate of oral contraceptive users was limited (4.5%), 43 of 59 students who used oral contraceptives to improve menstrual irregularity selected the items of Group A (regular) and Group B (constant, out of 26–32-day interval). On the other hand, 18 of 56 who used oral contraceptives to improve menstrual pain selected the items of Group I (free of pain) and Group II (not requiring analgesic). Consequently, to correct for the biases caused by the use of oral contraceptives, we statistically re-analyzed the relationship between dietary habits and menstrual disorders after the data of the oral contraceptive users were omitted, and obtained the same results. Even on transferring all data of 43 or 18 students in Groups I and II to Group III (irregular, but within 3 months) or Group III (painful, requiring analgesic), respectively, the statistical results were the same.

## 4. Discussion

This study showed that the incidence of troubles or worries with menstruation was significantly higher in those with breakfast skipping and a history of dieting. The aim of this study was to identify female students to whom health service centers should offer consultation and screening regarding the presence of psychological and/or physiological disorders of menstruation. Since more than a third of students experiencing troubles or worries with menstruation already visited the hospital, this simple questionnaire item is effective enough for initial screening by health service centers. Considering the small population hoping for consultation, further campaigns and intervention regarding menstrual disorders should be adopted on campus.

To analyze the reasons for troubles or worries with menstruation, we examined the relationship between menstrual disorders and dietary habits, and confirmed our previous reports that breakfast skipping is significantly correlated with dysmenorrhea [12,13]. Several studies reported similar findings [18,19,20]. By randomly selecting questionnaire samples from female Palestinian university students (*n* = 956), skipping breakfast was demonstrated to be the strongest predictor of moderate/severe dysmenorrhea using a visual analogue scale score [19]. In this study, the dietary habit of eating breakfast is classified into two groups: every day and sometimes/never. Recently, it was also shown that skipping breakfast was a risk factor of primary dysmenorrhea by analyzing Chinese female university students (*n* = 4606) [20]. This study defined those who did not eat breakfast one or more times in the past week as breakfast skippers (*n* = 3057, 69.0%). In contrast, another study demonstrated no significant correlation between the incidence of breakfast skipping and dysmenorrhea among female university students in Iran [21]. However, in this study, the definition of dysmenorrhea was different, and the numbers of dysmenorrhea-positive and -negative students were adjusted to the same ratio: *n* = 180 vs. 180. Furthermore, having breakfast was classified as normal if participants had breakfast one to six times per week and as low if having breakfast less than once a week. Despite this strict definition, the sample number and rate of the normal group were very low (*n* = 32, 8.9%), which may explain the reason for the discrepancy between our study and other studies. This suggests that the definition and interpretation of meal skipping should be considered based on local or national dietary habits.

In previous reports, we defined the stages of meal skipping as three groups: every day, one to six times a week, and less than once a week [13], whereas the third group was defined as “none” in this study. Since the populations that belonged to the group of “none” were very limited in the survey of lunch and dinner skipping, this definition may be too strict to analyze lunch and dinner skippers. Although we did not detect any significant correlation between dysmenorrhea and lunch or dinner skipping, meal skipping (one or two meal intake per day) was reported to be a risk factor for dysmenorrhea in 14–20-year-old women in Georgia (*n* = 2890) [22]. In contrast, the present study successfully detected a positive relationship between breakfast skipping and menstrual pain, indicating that this simple screening test is suitable for picking up breakfast skippers who are associated with gynecologic disorders at health service centers.

Both food intake and the light/dark cycle are key regulators of circadian rhythms regulated by the central clock system [23,24,25]. Since skipping breakfast interferes with the start of the active phase during the circadian rhythm, we proposed that the central clock system is involved in breakfast skipping-related dysmenorrhea [15]. In the adolescent stage, abnormal uterine contraction is usually caused by ovarian dysfunction [26], and it is one of main causes of primary dysmenorrhea [27,28]. Ovarian immaturity in steroid hormone production leads to intrauterine elevation of prostaglandins and leukotrienes during menstruation, which in turn induces excessive uterine contractions that are associated with pain [28,29]. Accordingly, we suggest that the disruption of the circadian rhythm caused by breakfast skipping affects the hypothalamic–pituitary–ovarian axis and impairs the reproductive rhythm, leading to ovarian dysfunction and dysmenorrhea [13,15]. In our animal experiments, when the timing of food intake in post-adolescent female rats was restricted only to the daytime (fed only in the non-active phase), ovarian functions were significantly disrupted as compared with those of rats fed during the active phase, providing supporting evidence that the timing of food intake can affect the function of the hypothalamic–pituitary–ovarian axis [30].

Considering that reproductive organs extensively develop, grow, and mature during adolescence and young adulthood, these periods are critical for establishment of the female reproductive function. Consequently, breakfast skipping during these stages may impair functional development and maturation of the reproductive organs, inducing the latent progression of obstetrics and gynecologic disorders [13,14]. Notably, abnormal uterine contraction can cause retrograde menstrual effluent flow into the peritoneal cavity, ectopic implantation of endometriotic lesions, and the onset of endometriosis, which will later manifest as dysmenorrhea [31,32]. Taking its importance into account, we recently proposed defining this pathological condition caused by inadequate dietary habits as ADHOGD [15]. Dysmenorrhea is one of the important clinical signs that suggest the presence of gynecological organic diseases together with ovarian dysfunction [33]. It should also be noted that breakfast skipping can be corrected by education. Accordingly, from a preventive standpoint, we propose that breakfast skipping in female college students is a potential predictor of gynecologic disorders, which can be screened for and prevented at health service centers.

Although the high incidence of troubles or worries with menstruation was observed in diet-experienced groups, the present study did not detect a positive relationship between a history of dieting and menstrual irregularity or menstrual pain. Preceding studies reported that about 40 to 60% of adolescents tried to lose weight and that dieting was closely associated with menstrual irregularity and pain [5,34]. In our previous studies, a history of dieting was defined as those who experienced a total reduction of more than 3 kg in body weight without restriction of its duration, showing that such a population made up about 60% [14]. In contrast, this study limited the duration to 3 kg reduction within a month, leading to a small population (6.1%). This strict limitation may define considerable numbers of students with a dieting history as those with no history, and explain the reason why this study failed to detect the effects of a history of dieting on menstrual disorders.

Our study has several limitations. First, this study is based on a single-year survey. The collection of long-term data should be performed to obtain more definite conclusions. Second, this study lacked the data of major confounders, such as the overall dietary intake, dietary patterns, and emotional or environmental factors, which can be related with dietary habits and worries with menstruation. Skipping meals might be related to lower intake of energy and some nutrients. Third, our Health Questionnaire Sheet does not contain the questions about body weight and height, because of ethical limitation, this study could not discuss the relationship among body mass index, dietary habits, and menstrual disorders.

## 5. Conclusions

This study showed that the incidence of troubles or worries with menstruation was significantly higher in those with breakfast skipping and a history of dieting. The present study also confirmed that female students who skip breakfast have a significantly higher incidence of dysmenorrhea, suggesting that breakfast skipping affects ovarian and uterine functions. Since dysmenorrhea is one of the important clinical signs, breakfast skipping may become an excellent marker to predict the subsequent onset of gynecological diseases at health service centers. To our knowledge, this is the first study to caution gynecologists that breakfast skipping is a key dietary habit that may induce dysmenorrhea-related obstetric and gynecologic diseases. Considering that meal skipping can be corrected by education, breakfast skipping is a potential and preventable predictor that may contribute to managing menstrual disorders from a preventive standpoint in the future.

## Figures and Tables

**Table 1:** Relationship between the incidence of troubles or worries with menstruation and meal skipping or dietary habits.

Having Meals	Without Troubles or Worries	With Troubles or Worries		
**Breakfast (n/%)**				
Every day	1756/83.6	345/16.4	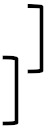	**
Sometimes	707/80.2	174/19.8		
None	98/76.6	30/23.4		
**Lunch (n/%)**				
Every day	2429/82.7	507/17.3	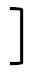	*
Sometimes	132/76.3	41/23.7		
None	0/0.0	1/100.0		
**Dinner (n/%)**				
Every day	2410/72.6	507/17.4	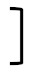	n.s.
Sometimes	150/78.5	41/21.5		
None	1/50.0	1/50.0		
**History of dieting (n/%)**				
Not done	2423/83.0	496/17.0	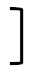	***
Done	138/72.3	53/27.7		
At present	9/75.0	3/25.0		
Past at 18<	59/72.0	23/28.0		
Past at 18≥	56/76.7	17/23.3		

***: *p* < 0.001, **: *p* < 0.01, *: *p* < 0.05, n.s.: not significant.

**Table 2:** Risks for menstrual problems related to dietary habits and dieting history.

	Trouble or Worries with Menstruation		Menstrual Pain	
	Odds Ratio (95% CI)	*p* Value	Odds Ratio (95% CI)	*p* Value
Having breakfast Everyday vs. Sometimes or none	1.22 (1.00–1.49)	0.048	1.23 (1.05–1.43)	0.012
Having lunch Everyday vs. Sometimes or none	1.36 (0.91–2.01)	0.124	1.29 (0.92–1.79)	0.137
Having dinner Everyday vs. Sometimes or none	1.00 (0.68–1.45)	0.966	0.94 (0.68–1.30)	0.697
History of dieting				
Done vs. Not done	1.88 (1.35–2.61)	<0.001	1.05 (0.78–1.42)	0.740

Outcome of pain was defined as “requiring analgesic” or “not requiring analgesic”. CI: confidence interval.

**Table 3:** Relationship between menstrual irregularity and meal skipping or dietary habits.

Meal Skipping	Regular	Constant Out of 26–32-Days	Irregular, within 3 M	Sometimes, More than 3 M		
**Breakfast (n/%)**						
Every day	1420/67.6	381/18.1	255/12.1	45/2.1	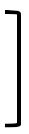	n.s.
Sometimes	594/67.4	162/18.4	112/12.7	13/1.5		
None	77/60.2	35/27.3	12/9.4	4/3.1		
**Lunch (n/%)**						
Every day	1971/67.1	551/18.8	353/12.0	61/2.1	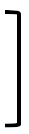	n.s.
Sometimes	119/68.8	27/15.6	26/15.0	1/0.6		
None	1/100.0	0/0.0	0/0.0	0/0.0		
**Dinner (n/%)**						
Every day	1974/67.7	535/18.3	349/12.0	59/2.0	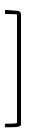	n.s.
Sometimes	116/60.7	43/22.5	29/15.2	3/1.6		
None	1/50.0	0/0.0	1/50.0	0/0.0		
**History of dieting (n/%)**						
Not done	1973/67.6	539/18.5	352/12.1	55/1.9	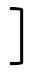	n.s.
Done	118/61.8	39/20.4	27/14.1	7/3.7		
At present	10/83.3	1/8.3	0/0.0	1/8.3		
Past at 18<	51/62.2	18/22.0	12/14.6	1/1.2		
Past at 18≥	49/67.1	11/15.1	9/12.3	4/5.5		

M: months, n.s.: not significant.

**Table 4:** Relationship between menstrual pain and meal skipping or dietary habits.

Meal Skipping	Free of Pain	Not Requiring Analgesic	Requiring Analgesic	Not Relieved by Analgesic				
Breakfast (n/%)								
Every day	496/23.6	822/39.1	746/35.5	37/1.8	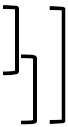	n.s.		***
Sometimes	169/19.2	354/40.2	343/38.9	15/1.7			***	
None	18/14.1	38/29.7	68/53.1	4/3.1				
**Lunch (n/%)**								
Every day	650/22.1	1153/39.3	1083/36.9	50/1.7	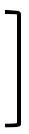	n.s.		
Sometimes	33/19.1	61/35.3	73/42.2	6/3.5				
None	0/0.0	0/0.0	1/100.0	0/0.0				
**Dinner (n/%)**								
Every day	635/21.8	1148/39.4	1083/37.1	51/1.7	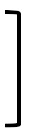	n.s.		
Sometimes	48/25.1	65/34.0	74/38.7	4/2.1				
None	0/0.0	1/50.0	0/0.0	1/50.0				
**History of dieting (n/%)**								
Not done	641/22.0	1143/39.2	1086/37.2	49/1.7	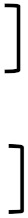	n.s.		
Done	42/22.0	71/37.2	71/37.2	7/3.7				
At present	4/33.3	4/33.3	3/25.0	1/8.3				
Past at 18<	18/22.0	37/45.1	25/30.5	2/2.4		n.s.		
Past at 18≥	17/23.3	22/30.1	31/42.5	3/4.1				

***: *p* < 0.001, n.s.: not significant.

**Table 5:** Relationship between use of contraceptives and meal skipping or dietary habits.

Meal Skipping	Not Using	Using for Menstrual Cycle	Using for Menstrual Pain	Other Reasons		
Breakfast (n/%)						
Every day	2012/95.8	41/2.0	33/1.6	15/0.7	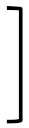	n.s.
Sometimes	837/95.0	15/1.7	21/2.4	8/0.9		
None	121/94.5	3/2.3	2/1.6	2/1.6		
**Lunch (n/%)**						
Every day	2805/95.5	55/1.9	53/1.8	23/0.8	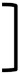	n.s.
Sometimes	164/94.8	4/2.3	3/1.7	2/1.2		
None	1/100.0	0/0.0	0/0.0	0/0.0		
**Dinner (n/%)**						
Every day	2785/95.5	56/1.9	52/1.8	24/0.8	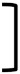	n.s.
Sometimes	184/96.3	3/1.6	3/1.6	1/0.5		
None	1/50.0	0/0.0	1/50.0	0/0.0		
**History of dieting (n/%)**						
Not done	2798/95.9	55/1.9	47/1.6	19/0.7	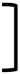	***
Done	172/90.1	4/2.1	9/4.7	6/3.1		
At present	9/75.0	0/0.0	2/16.7	1/8.3		
Past at 18<	74/90.2	2/2.4	4/4.9	2/2.4		
Past at 18≥	66/90.4	2/2.7	2/2.7	3/4.1		

***: *p* < 0.001, n.s.: not significant.
